# Oncolytic bacteria: A revolutionary approach to cancer therapy

**DOI:** 10.1515/biol-2025-1076

**Published:** 2025-06-10

**Authors:** Mohsen A. Khormi, Saleh M. Al-maaqar, Abdulaziz R. Al Johni, Nasser A. Al-Tayyar, Jafar Abdullah Alhamad, Abdullah A. Ghyathuddin, Zakia Alblawi, Sabreen M. Behairy, Mohammed A. Alghamdi, Wael A. Alsubhi, Mikiyas D. Teklemariam

**Affiliations:** Department of Biology, College of Science, Jazan University, P.O. Box 114, Jazan, 45142, Saudi Arabia; Department of Biology, Faculty of Education, Albaydha University, Al-Baydha, Yemen; Department of Biological Sciences, Faculty of Science, King Abdulaziz University, P.O. Box 80203, Jeddah, 21589, Saudi Arabia; Department of Laboratory at King Fahad Hospital, Madinah, Saudi Arabia; General Directorate of Education in Jeddah, Madinah, Saudi Arabia; Department of Pharmacy Practice, University of Hafr Al Batin, Hafr Al Batin, Saudi Arabia; School of Nursing, Department of Midwifery, Addis Ababa University, College of Health Sciences, Addis Ababa, Ethiopia

**Keywords:** cancer, oncolytic bacteria, immunity, genetically modified bacteria

## Abstract

Cancer is one of the most devastating diseases all over the globe, and it is the second worldwide cause of death, exceeded only by cardiovascular diseases. The therapeutic approach to human cancer has evolved significantly and has varied depending on the type and stage of cancer, as well as the general health status of the patient. Despite the advancements in cancer treatment, various challenges persist in the treatment of cancer, including side effects, drug resistance, and incomplete eradication of tumors. The use of oncolytic bacteria (cancer targeting and destroying bacteria) has been identified to have several advantages over the traditional methods of cancer treatment. Several bacterial species have been identified to be used in the treatment of different types of cancers. Oncolytic therapy can be achieved through the use of a naturally occurring and/or genetically modified bacterial species, including *Clostridium*, *Salmonella, Escherichia coli,* and *Listeria* spp. with their toxins, enzymes, biofilms, and secondary metabolites as well as their spores that leads to direct or indirect killing of cancer cells. This review provides some highlights about the biology and therapeutic potential of oncolytic bacteria individually or in combination with other therapeutic approaches against different types of cancers. Besides, the current challenges and future perspectives will be explored.

## 1 Introduction

Cancer is one of the most devastating diseases worldwide, and according to Siegel et al. [1], it ranks as the foremost cause of death worldwide. Characteristics such as angiogenesis, immortalization, uncontrolled and deregulated cell growth, invasion, and metastasis are some of the hallmark features of cancer [2]. A total of 19,976,499 cases of cancer were diagnosed in 2022. In the same year, the age-standardized rate for all cancers for women and men combined was 196.9 per 100,000. The rate was lower for women (186.3 per 100,000) than men (212.6 per 100,000) (https://www.wcrf.org/preventing-cancer/cancer-statistics/global-cancer-data-by-country/ accessed on December 18, 2024). In 2023, a total of 80,502 malignant tumors affecting 73,794 people were reported to the cancer register [3]. In 2024 alone, 2,001,140 new cancer cases and 611,720 cancer fatalities are anticipated to occur in the United States [4]. Lung cancer is one of the most frequently diagnosed and accounts for nearly 1.8 million death tolls annually. Breast cancer comes next and is responsible for around 2.3 million cases annually, rating it the most prevalent among women than men. Another major cancer that is frequently encountered in humans is colorectal cancer (CRC). CRC accounts for 1.9 million cases and is accompanied by 900,000 deaths annually. Besides, prostate cancer is the other prevalent cancer type in men, resulting in 1.5 million new cases annually (https://www.who.int/news-room/fact-sheets/detail/cancer accessed on December 19, 2024). Due to rapid inclination in the population size, the burden of cancer is expected to be projected significantly, exceeding by over 60% by 2040. This means that the number of new cancer cases is anticipated to grow from 18.1 million in 2018 to an estimated 29.4 million cases by 2040 (https://canceratlas.cancer.org/the-burden/the-burden-of-cancer/ accessed on June 27, 2024).

Cancer is a disease that has multiple causes, including genetic, environmental, and ones that are lifestyle-related [5,6]. The root cause of cancerous growth is the mutations of certain genes that regulate the cell’s growth and division and may cause cells to divide uncontrollably in an abnormal manner. Radiation and carcinogenic chemicals, which people are exposed to through their work environment, increase the risk [7,8]. Other predisposing factors include smoking, alcohol consumption, having an improper diet, having a sedentary lifestyle, and obesity [9]. Besides, inflammation and hormonal changes also contribute to the growth of cancer. Infectious diseases also account for a significant case of cancer worldwide [10]. These comprised infections caused by *Helicobacter pylori* (linked to stomach cancer), human papillomavirus (cervical and other cancers), hepatitis B and C viruses (liver cancer), and Epstein–Barr virus (lymphoma). The multifactorial cause of cancer imposes thorough preventive approaches that address most of the risk factors linked to it [11].

According to documented reports, the therapeutic approach to human cancer has evolved significantly and has varied depending on the type and stage of cancer, as well as the general health status of the patient [12,13]. The major treatment approaches include surgery, radiation, chemotherapy, immunotherapy, molecular therapy, and hormonal therapy. In the case of the surgical approach, the tumor is mechanically cut out and is best suited for solid cancers. Radiation therapy is a course of high-energy rays to destroy cancer cells and is sometimes recommended along with surgery [14]. Immunotherapy helps the body to enhance the immune system to fight cancer, while targeted therapy aims at targeting certain proteins that lead to the development of cancer [15]. Selective hormonal-based therapy is another form of therapy employed in hormone receptor-positive cancers, for instance, breast and prostate malignancies [16]. Precision medicine has also facilitated the emergency of personalized cancer treatment, where therapies rely on molecular and genetic features of the tumor cell [17].

Despite the recent advancements in cancer treatment, several limitations have been documented. Therapeutic failure is one of the most alarming challenges of cancer therapy, which occurs when cancer cells evolve and become resistant to treatments such as chemotherapy and targeted therapies. Moreover, the adverse effects of chemotherapeutic agents can complicate the therapeutic outcome and may expose patients to other chemotherapy-induced diseases. Another major challenge is the accessibility and cost of emerging treatments, which can be expensive and mostly available in high-income countries [18]. Also, there is a need for improvement in the diagnostic modality concerning cancer with a view to increasing the chances of diagnosing cancer at an early stage.

Tumor heterogeneity is known to be a detrimental factor that influences the treatment of cancer. It is a phenotypic, genetic, and/or behavioral variation of the cancer cells, which may occur within a single cancer cell [19]. It can occur through epigenetic, genetic, and protein modifications. Heterogeneity provides tumors with notable resilience and may affect the levels of their response to a given treatment [20].

Because of remarkable survival capability and high mutagenic ability, cancer cells utilize many strategies to evade the host immune activity to maintain their proliferation and sustain progress [21]. Main evasion strategies include triggering the production of suppressive cytokines such as TGF-β and IL-10, upregulation of checkpoint receptor ligands that potentially prevent tumor-infiltrating lymphocytes from uniting the mass of the tumor, or upregulation of immune-suppressing cells, including regulatory T-cells (Tregs) [22]. Other strategies encompass downregulating the components of the antigen presentation system [22].

The development of the tumor microenvironment (TME) is the other evasive mechanism that enables the tumor cell to develop but facilitates the recruitment of host immune system components. The structural integrity of the TME mainly acts as a mechanical barrier to stop the infiltration of the immune cells [22,23]. The development of a thick stromal layer surrounding the cancerous mass creates a mechanical barrier that is characterized by several characteristics proven to facilitate the growth of cancer, including the creation of a hypoxic environment and deviant tumor neovascularization [24]. Another factor that hampers the treatment of cancer involves individual patient variation in response to treatments that are also in part due to the side effects of conventional therapies, making it crucial to explore new treatments that are less toxic for the human body [25].

The use of oncolytic bacteria, which is cancer targeting and destroying bacteria, has been identified to have several advantages over the traditional methods of cancer treatment. Several bacterial species have been identified to be used in the treatment of different types of cancers. According to research findings, oncolytic therapy can be achieved through the use of a naturally occurring and/or genetically modified (GM) bacterial species, including *Clostridium*, *Salmonella*, *Escherichia coli,* and *Listeria* spp. with their toxins, enzymes, biofilms, and other secondary metabolites as well as their spores that leads to direct or indirect killing of cancer cells [26].

Oncolytic bacteria can reach hypoxic areas of tumors in which other forms of therapy can be ineffective. The reproduction potential of oncolytic bacteria at the hypoxic region is very crucial to generating lifelong therapeutic effects. Besides, oncolytic bacteria are able to activate the host’s immune system against cancer cells, thus maximizing the overall anticancer process [27].

The aim of this review is to describe oncolytic bacteria in terms of how they work, various issues associated with their use, and prospects for their future application. It explains the major oncolytic strategies, such as toxin production, biofilm formation, and immune response stimulation. In addition, it will also discuss the existing challenges and barriers associated with the clinical use of bacterial therapies, such as the problem of toxicity, genetic stability, and immune modulation. Finally, the review will discuss potential perspectives for the improvement of the safety and efficacy of oncolytic bacterial therapy as well as the possibilities of combination therapy with other treatment modalities.

## 2 What are oncolytic bacteria?

Oncolytic bacteria are those bacteria that target and destroy only cancer cells without harming the other cells of the body. These bacteria can be native or GM to improve their tumor-homing properties. After reaching inside the tumor, oncolytic bacteria are capable of killing tumor cells, mainly by bacterial infection, stimulation of immune cells, or by the liberation of toxic material. The use of bacteria for cancer therapy was initiated in the late nineteenth century by William B. Coley, who introduced the concept of “Coley’s toxin,” a mixture of heat-inactivated bacteria that displayed efficacy in curing sarcomas [28].

Currently, the emergence of more selective and effective oncolytic bacteria is partly linked to the advancement of new techniques in the realm of genetic engineering. The genetically engineered (GE) bacteria can be designed to generate therapeutic genes, release therapeutic biomolecules that modulate the immune system or minimize potential side effects. For example, some strains such as *Salmonella typhimurium* have been GM to improve their ability to target tumors and to eliminate pathogenicity and they have shown good results in preclinical and clinical trials [29]. Currently, the field of oncolytic bacterial therapy is still progressing with more research works conducted to enhance bacterial strains in the treatment of cancer with better safety and efficacy [30].

## 3 Historical background

The application of bacteria in the treatment of cancer dates back to the nineteenth century. In 1867, German physician Wilhelm Busch described a case of a cancer patient whose tumor size decreased after a severe infection with erysipelas, now known as *Streptococcus pyogenes* [31]. In the late nineteenth century, William B. Coley visualized head and neck cancer patients displaying substantial remission following infections [31]. Based on some of the patients’ records, he developed what he called “paradoxical” cancer treatment through intravenous administration of heat-inactivated *Serratia pyogenes* and *Serratia marcescens* known as Coley’s toxin. During the subsequent 40 years, a variety of sick people displayed various degrees of remission from sarcoma employing “Coley’s toxin,” with 10-year survival rates similar to those of individuals who received contemporary conventional treatment. This success earned Coley the title “the father of cancer immunotherapy.” His observations sparked research into bacterial-based cancer therapy and paved the way for a new era of cancer treatment [32].

Kim et al. investigated immune responses by examining the cytokine constituents involved and the population of immune cells within tumors infected with either the *E. coli* MG1655 strain or the attenuated *S*. t*yphimurium* ΔppGpp (guanosine 5′-diphosphate-3′-diphosphate) strain. They found that interleukin-1β (IL-1β) and tumor necrosis factor-α (TNF-α) were primarily secreted by dendritic cells (DCs), and macrophages with a marked elevation in these cytokines in tumors colonized by ΔppGpp all through the phase of tumor suppression. This suggests that TNF-α and IL-1β may play crucial roles in the anticancer effects mediated by *Salmonella* [33].

## 4 Mechanisms of action

### 4.1 Anti-tumor immune responses (immunosuppression)

The induction of anti-tumor immune responses by oncolytic bacteria is an emerging and promising area of cancer research. Oncolytic bacteria are either modified or non-modified bacteria that selectively kill cancer cells [34,35]. These bacteria can directly kill the cancer cells and destroy them, which leads to the release of tumor-associated antigens, thus provoking a higher degree of immune response from the body. This process not only assists in the depletion of the tumor mass but also assists in the generation of long-term anti-tumor immunity by stimulating innate immunity as well as adaptive immunity. Consequently, the body’s immune system is prepared to target the remaining cancer cells to possibly reduce the chance of recurrence [36].

The process through which oncolytic bacteria stimulate anti-tumor immune responses is a multi-step process. First, the bacteria selectively adhere to the TME, which can be hypoxic and immunosuppressive, whereby conventional treatment is also ineffective. In this case, bacteria that are capable of accessing the site within tumor tissues can produce toxins or enzymes that result in the killing of tumor cells [37]. The constant secretion of tumor-associated antigens to the extracellular fluid of the tumor increases the immigration and activation of DCs. These DCs then take these antigens to internalize, process, and present them to T cells, triggering a specific adaptive immune response. This chain of events not only affects the primary tumor but also affects the progression of metastatic cancer cells [38].

According to the documented findings, current clinical and preclinical research proves the efficacy of oncolytic bacteria in the case of several types of cancers: melanoma, CRC, and glioblastoma [39,40]. In such research, investigators have revealed that oncolytic bacteria can be administered singly or together with other treatments, including immune checkpoint inhibitors, to improve the treatment outcomes. This attribute of oncolytic bacteria is to reshape the TME and recruit the immune system to the tumor site while also overcoming mechanisms that enable tumor immune evasion [40,41]. With growing research in the sector, oncolytic bacteria can effectively complement the growth of cancer immunotherapy and give fresh hope to patients with both refractory and recurrent cancers.

The key components of the immune system that have a significant impact on suppressing malignant and deformed cells associated with bacteriotherapy are macrophages, CD8+ T-lymphocytes, DCs, natural killer (NK) cells, and T-regs, which contain forkhead box P3 (FOXP3) as a biomarker. These cells have a significant impact on the propagation of cancerous cells and destruction as well as eradicating them [42].

**Figure 1:**
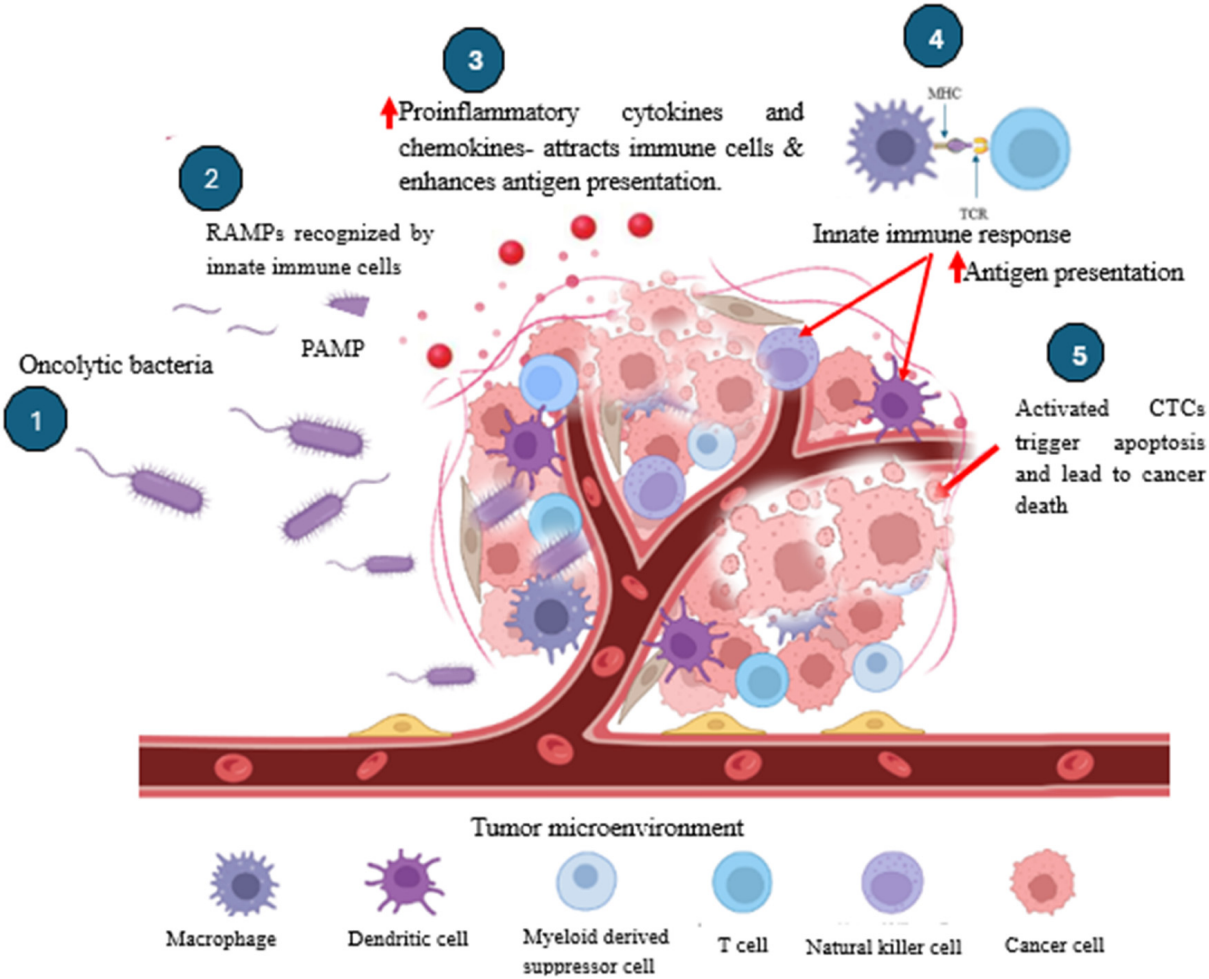
A schematic diagram illustrating the roles of innate immune response and acquired immune response during bacteria-mediated cancer therapy. Created and modified with BioRender.com.

Bacteria-induced infections (e.g., *E. coli*) can be employed to inhibit the growth of tumor tissues. These induced infections enhance and facilitate the differentiation of CD8+ killer T-cells, giving rise to the synthesis of IFN-γ and promoting the expression of major histocompatibility complex subtype I (MHC-I) on cancerous cells. This integrative strategy led to the targeted destruction of tumor cells, enhancing the ability of the immune system to recognize and eliminate malignant tissues (Figure 1) [43]. Tumor necrosis factor (TNF-*α*) also contributes to the formation of drainage in tumors. The bacterium is able to stimulate the production of tumor necrosis factor, which has been reported with *S. typhimurium* [44].

### 4.2 Direct tumor cell lysis

The oncolytic bacteria use various strategies to hasten the growth of cancer cells. It has been known that direct killing of the cancer cell is one of the methods employed (Table 1) [45]. Oncolytic bacteria can directly colonize cancer-harboring tissue and they deploy their toxins that disrupt the functions of the attacked cells and enzymes that lyse the targeted cells. For instance, *Clostridium novyi* -NT produces alpha-toxin that has the ability to affect and break cell membranes, thereby causing cell rupture and death. Another one is based on using the *S*. *typhimurium* bacterium that synthesizes bacterial proteins, causing the apoptosis of tumor cells. These bacteria have a predilection for the tumor microenvironment, which, as we know, is characteristically hypoxic and pro-angiogenic and lacks nutrient supply for bacterial growth and function. This selective targeting reduces the probability of harming other adjacent tissues and organs while enhancing the anti-tumor outcomes [46]. *Salmonella* also has enviable characteristics due to its capability to propagate within the cancer cells and promote regression and death [47,48].

**Table 1:** Summary of studies on the anticancer effects of various bacterial species/strains

Bacterial species/strains	Cancer type	Mechanism of action and results	References
*L. monocytogenes*	Renal cell carcinoma (RCC)	Generate a significant anti-tumor efficacy in both subcutaneous and orthotopic RCC tumor models by influx of tumor-infiltrating effector T cells. It has also attracted a broader spectrum of polyfunctional T cells into the TME and trigger resilient IFN-γ response	[61]
Oncolytic bacteria VNP20009	Melanoma	Expressing IFNβ that inhibits the progression of melanoma by remodeling the TME. In comparison to VNP, VNP-IFNβ mobilized more macrophages and neutrophils with anti-tumor phenotypes in lung metastases and activated DCs differentiation, which stimulated the anti-tumor immune responses of CD4^+^ T cells, and finally the progression of melanoma inhibited	[39]
*Lactobacillus crispatus* strains Lc31 and Lc83	Cervical cancer	The availability of genes encoding for bacteriocins in the urogenital strains coupled with the lack of virulence factors is suggested to be the possible mechanism of action for its anticancer probiotic effects	[62]
*Shigella flexneri*	Pancreatic cancer	The organism displayed an anti-proliferative effect (upregulate Bax and downregulate bcl-2)	[63]
*Bacillus coagulans*	Breast cancer	Induction of apoptosis	[64]
*Bifidobacterium bifidum* and *Bacteroides fragilis*	Breast cancer	Through inhibiting angiogenesis and proliferation and induction of necrosis	[65]
*Lactobacillus brevis* MK05	Breast cancer	Cytotoxic activity and induction of apoptosis	[66]
*B. longum C-CPE-PE23*	Breast cancer	Tumor growth suppression in mice	[67]
*Bacterium L. reuteri FLRE5K1*	Hepatocellular carcinoma	Inhibiting the progression of the tumor by the induction of the IFN-γ/CXCL10/CXCR3 pathway	[68]
*Bifidobacterium* species	Colon cancer	Downregulated the expression of HER-2, EGFR, and PTGS-2 (COX-2)	[69]
*Lactobacillus acidophilus* NCFM	Colon cancer	Inhabit the expression of MHC class I and CXCR4 mRNA. Enhance apoptosis in tumor cells	[70]
*C. novyi*	Breast cancer	*C. novyi* types A and B secreted alpha toxins that damage the hypoxia zone, and the aerobic compartment of the breast tumor was regressed fully (achieved 100% eradicate breast tumors in mice where the size of the tumor reduced to as small as 1,000 mm^3^ tumors))	[71]
*Clostridium ghonii*	Experimental solid tumors	*C. ghonii* secreted phospholipase c and collagenase IV, improved necrosis and apoptosis in cultured A549 cells. Spores germinated in the tumors and induced immune activation following intratumoral injection in tumor-bearing mice. The spores of this organism reduced the volume of the tumor and promoted tumor necrosis	[72]
dL5**-expressing *S*. *typhimurium* strain	Colon cancer	Enhancing the anticancer characteristics by pathogen-linked molecular patterns activated immune cells and minimized anti-inflammatory cytokines (IL-10) enhancing anticancer effects	[73]
Engineered *S*. *typhimurium* expressing ClyA	Pancreatic cancer	Following the intravenous administration of the agent (attenuated bacteria), it is accumulated and proliferated. ClyA is secreted and released into the TME. Histological analysis indicated a notable decline in the expression of markers of the stromal cell and potentiated immune cell (macrophages and neutrophils) infiltration into tumors	[74]

Bacteria can generate anti-tumor effects via the depletion of nutrients essential for the metabolism of cancer cells [49]. The tumor tissues that are deoxygenated (anoxic region) promote the aggregation of obligatory anaerobic bacteria in the region [50]. Research has demonstrated that the systemic introduction of *Salmonella* bacteria disseminated toward the solid tumor via the hemorrhaging area inhabited by the tumor exerted pressure and minimized the proliferation of the tumor [51]. The necrotic regions are formed due to the reduction of nutrient and oxygen supply, which leads to the breaking down of the viable blood vessels, which in turn causes the death of the tumor cells from suffocation and starvation [51].

Yam et al. (2010) demonstrated that a genetically manipulated auxotroph strain of *S. typhimurium* was able to selectively and effectively suppress liver metastasis in a mouse model of pancreatic cancer. To increase tumor-targeting ability and tumor-killing efficacy, the *S. typhimurium* strain was additionally modified by re-isolation from a tumor growing in a nude mouse. The strain obtained from this method was termed A1-R. In this study, the authors explored the efficacy of locally as well as systemically introduced A1-R on liver metastasis of pancreatic cancer. Mice who received A1-R locally via the intrasplenic route of administration or systemically via tail vein injection experienced a notable reduction in splenic and hepatic tumor burden relative to the control group. Intravenous injection of A1-R also improves survival time of the treatment group [52].

Some bacterial species, like *E. coli,* exhibit tropism for tumor tissue, which secretes chemical constituents such as clusterin, TGF-β2, and serglycin, while others, like facultative anaerobes (e.g., *Escherichia* and *Salmonella*), can sense the nutrient-rich environment of tumors and contribute to their accumulation in the tumor cells and exert their killing effect [53,54].

*Listeria* infection can directly kill tumor cells via the propagation of nicotinamide adenine dinucleotide phosphate (NADP+) oxidase and elevated levels of intracellular Ca^2+^ [55]. These mechanisms can generate cytotoxic free radical, reactive oxygen species (ROS), [56] which can promote the immunogenic death of tumor cells, then activate CD8+ T cells to clean up residual tumor cells and ultimately avert metastases. Additionally, *Listeria* can infect bone marrow-derived suppressor cells (MDSCs) at the tumor site, causing a significant reduction in MDSCs and, consequently, transforming the immunosuppressive microenvironment to an immunostimulatory condition, which can maximize the response of T cells and NK cells toward the cancer cells [57].

*Clostridium* kills the tumor cells mostly by producing some exotoxins following its colonization. For example, phospholipases and hemolysins can destroy tumor cells by attacking their membrane [58]. This organism can promote the apoptosis of tumors by activating the release of tumor necrosis factor-related apoptosis-inducing ligands from polymorphonuclear neutrophils. Furthermore, the early expansion of *Clostridium* in solid tumors could lead to intratumoral infiltration of macrophages and granulocytes and maximize the secretion of chemokines, which might provoke the adaptive immunity and recruit immune cells to the tumor site and facilitate the killing process [59].

According to Qi et al., *P. aeruginosa* 1409 triggered programmed necrosis (necroptosis) of TC-1 tumor cells through activation of TLR4-RIP3-MLKL and the HMGB1 released by the dying tumor cells. According to their report, such a mechanism reshaping the TME leads to remarkable tumor suppression in a TC-1-grafted tumor mouse model [60].

### 4.3 Microbial biosurfactants

Biosurfactants are chemical compounds that have both hydrophilic (polysaccharides, amino acids, peptides, and various ions) and hydrophobic (saturated or unsaturated fatty acids) properties. These compounds are known for various physiological activities, including their capability to minimize the tension exerted on the surface and interfacial between liquids [75]. Biosurfactants are mainly classified into low- and high-molecular-weight compounds. The low-molecular-weight compounds consist of fatty acids, lipopeptides, lipoproteins, phospholipids, and glycolipids, while the high-molecular-weight compounds of biosurfactants contain polymeric biosurfactants [76].

The anticancer potential of biosurfactants is attributed to their ability to hinder cancer cell growth, enhance apoptosis, trigger necrosis, and arrest the cell cycle [77,78]. These biosurfactants comprise surfactin [79], mannosylerythritol lipids (MELs) [80], sophorolipids (SLPs) [81], rhamnolipids (RLs), succinoyl trehalose lipids (STLs) [82], serrawettins [83], and monoolein [84].

Biosurfactants are chemicals that exhibit surface activity and are synthesized by various microorganisms, including bacteria, fungi, yeast, and other small algae [85]. These compounds are recognized by their ability to lower the surface and interfacial tension, rendering them useful in many sectors and processes. More recently, they have been reported to possibly act as anticancer agents because of their characteristics and action modes. In addition, biosurfactants possess several biological activities such as antimicrobial, antiviral, and anti-inflammatory properties that endow them with anticancer properties. They are able to provoke apoptosis, inhibit the spread of cancer cells, and increase the effectiveness of initial chemotherapy. Also, biosurfactants have been found to possess properties that enable them to preferentially affect cancer cells without harming normal cells, and due to this, there are fewer side effects when treating cancer. In addition, the biodegradability and low toxicity of these molecules add to the rationale for the development of such agents as therapeutics. Studies continue in an attempt to understand all aspects of their anticancer potential and the preparation of biosurfactant-based products for clinic use as the next horizon in cancer therapy [86].

Several biosurfactants produced by different bacterial strains, such as RLs, SLPs, and MELs, have shown promising therapeutic activities against cancer cells. RLs synthesized by *P. aeruginosa*, for instance, have a cytotoxic effect on different cancer cell lines, including breast cell lines (MCF-7, MCF-10A, and MDA-MB-231) [87], acute leukemia cells (SKW-3, BV-173), promyelocytic leukemic cells (HL-60), and murine cells. In one of the studies, RLs were found to exert a substantial effect in causing cytotoxicity to CRC cell lines, including HCT-116 and Caco-2, but the non-significant effect to normal cells, indicating that their selectivity was inclined toward the cancer cells [88]. In another study, Mishra et al. [89] investigated the properties and apoptotic effects of bacterial RLs on MDA-MB-231 breast cancer cells. Their research shed light on the fact that such RLs exert their antiproliferative action by targeting and modulating the p38 mitogen-activated protein kinase (MAPK) signaling pathway, which plays crucial roles in cell proliferation and survival.

Lipopeptide, surfactin in particular, which is a microbial metabolite of *Bacillus subtilis,* is a potent anticancer agent that has been found to trigger apoptosis and restrain metastasis in numerous cancer cells. Surfactin suppresses cell viability through the modulation of membrane fluidity and the induction of apoptosis through the caspase 3 and caspase 9 pathways [90]. Similarly, Zhao et al. [91] observed that lipopeptides from *B. subtilis*, mainly containing iturin, possess pronounced anticancer activity to chronic myelogenous leukemia cells (K562 cells) *in vitro*. The results revealed that these lipopeptides are capable of directly causing both apoptosis and paraptosis and, at the same time, suppressing autophagy. This multiple strategy highlights their promising role in the management of leukemia [91].

Studies revealed that a novel SLP, specifically C18:1 lactonic SLP (produced by *Starmerella bombicola*), showed enhanced cytotoxicity against the MDA-MB-231 breast cancer cell line. These SLPs inhibited cell migration without compromising cell viability. Furthermore, SLPs triggered the increase of intracellular ROS, which can cause stress and potentially damage cancer cells [92]. Similarly, Duarte et al. [93] showed the relevant effect of SLP against cancer cell growth in a dose-dependent manner. This was again associated with the induction of apoptosis due to a ROS and c-Jun N-terminal kinase (JNK)-mediated mechanism. This report supports that ROS is produced during the inhibition of cancer growth through SLP, producing an activation in JNK, inducing apoptosis. Table 2 provides different types of biosurfactants produced by bacterial species/strains and their anticancer effects.

**Table 2:** Summary of biosurfactants produced by bacterial species/strains and their anticancer effects

Bacterial species/strains	Biosurfactants	Cancer type	Mechanism of action and/or results	References
*Bacillus* sp. CS30	Surfactin CS30-2	Huh7.5 human liver cancer	Caused death of cancer cells via necrosis instead of apoptosis	[94]
*Enterococcus faecium*	Glycolipoprotein biosurfactant	Breast cancer cell lines (MCF-7)	Reduced the viability of the MCF-7 cancer cell line with a maximum inhibition percentage of 74.2% at a 100 µg/ml concentration	[95]
*P. aeruginosa*	Mono-RLs	CRC cell lines (Caco2 and HCT-116)	Results in cytotoxicity to the CRC cells	[88]
*B. subtilis* 573 and *Lactobacillus paracasei* subsp. *paracasei* A20	Surfactin and glycoprotein (BioEG), respectively	Breast cancer cell lines (MDA-MB-231 and T47D)	Surfactin reduced the viability of both breast cancer cell lines. A 24 h exposure to 0.05 g l^-1^ surfactin caused inhibition of the proliferation of cancer cells as displayed by cell cycle arrest at the G1 phase	[93]
*Bacillus tequilensis* CH and *Lysinibacillus fusiformis* S9	Biosurfactants CHBS and S9BS, respectively	Human embryonic kidney cancerous cell (HEK-293)	The biosurfactants demonstrated considerable cytotoxic effects on the HEK-293 cell line. The LC_50_ values of CHBS and S9BS were 100 and 75 μg ml^−1^, respectively. The cytotoxic effect of these glycolipids may be associated with the increased membrane permeability of biosurfactant-treated cancer cells due to the integration of its lipid moiety into the plasma membrane, resulting in pore formation and membrane destabilization	[96]
*Planococcus maritimus* SAMP MCC 3013	RL	MCF-7, cervical cancer (HeLa) and HCT	RL found to be cytotoxic against MCF-7 (IC_50_ 42.79 ± 6.07 μg/mL), HeLa (IC_50_ 41.41 ± 4.21 μg/mL), and HCT (IC_50_ 31.233 ± 5.08 μg/mL)	[97]
*Lactobacillus plantarum* 60 FHE	Glycolipoprotein biosurfactant	Colon carcinoma cells	The GP produced by *Lactobacillus plantarum* 60 FHE displayed anticancer activity against colon carcinoma	[98]
*P. aeruginosa* RA5	Di-rhamnolipids (Di-RLs)	Colon cancer (HT-29), MCF-7, leukemia (K-562), lung cancer (HOP-62), and HeLa	Di-RLs caused an inhibitory effect on the K-562 cell line with TGI and GI50 at 66.6 µg/mL and < 10 µg/mL, respectively, after 48 h of application	[99]
*B. subtilis* KLP2015	Surfactin lipopeptide (LP)	Human epithelial cell line (L-132), human colorectal adenocarcinoma cell line (HCT-15), human laryngeal carcinoma (Hep2-C), MCF-7, and mouse embryonic fibroblast cell line (NIH/3T3)	A fivefold reduction in DNA constituent was observed in LP-treated L-132 cells while twofold in Hep2-C after 20 h. However, SDS-treated Hep2-C and L-132 cells displayed nearly 1.4- and 1.5-fold reductions in DNA content from their initial content	[100]
*Acinetobacter* M6 strain	Not specified	Lung cancer cells (A549)	Anti-proliferative activity against A549, with a considerable reduction, demonstrated at 200 µg/ml concentration, caused inhibition of cell proliferation at the G1 phase	[101]
Marine *Bacillus circulans* DMS-2	Purified marine lipopeptides (PML)	Human colon cancer cell lines HT-29 and HCT-15	PML demonstrated significant antiproliferative activity against the human colon cancer cell lines HT-29 and HCT-15 at IC_50_ values of 120 μg ml^−1^ and 80 μg ml^−1^, respectively	[102]
*Pseudomonas gessardii*	Surfactin C15	HT-29 CRC cells	Surfactin C15 caused a significant anticancer activity involving multiple modes of action: downregulation of the expression of cancer stem cell marker CD133, upregulation of pro-apoptotic factors (PUMA, CHOP, and DR5), and modulation of regulators of the cell cycle (CCNE1, CDK5, and CDKN1A). Additionally, surfactin C15 triggered necrotic cell death, validated by increased release of lactate dehydrogenase and increased necrotic cells observed via flow cytometry analysis	[103]
*Acinetobacter junii (AjL) B6*	Lipopeptide biosurfactant (LPB)	Human glioblastoma cell line (U87), human epithelial carcinoma cell line (KB), and human umbilical vein endothelial cells (HUVECs)	LPB resulted in cytotoxicity against all cell lines at IC_50_ values of 2.4 ± 0.5 mg/ml, 7.8 ± 0.4 mg/ml, and 5.7 ± 0.1 mg/ml against KB, U87, and HUVEC cell lines, respectively	[104]

### 4.4 Bacterial spore

Recent studies reveal that bacterial spores, especially those belonging to the *Bacillus* group, are beneficial to cancer treatment [105]. Bacterial spores are very crucial structural and biochemical organizations of prokaryotes that protect the agents under extremely unfavorable conditions (extreme temperatures, radiation, and desiccation). This ability to withstand unfavorable conditions, together with their capacity to stay dormant until conditions improve, makes them a favorable candidate for many uses in the medical field, especially in cancer research. Studies have demonstrated that bacterial spores can home in on cancer cells and kill them with minimal effects on the healthy cells. This selective targeting is mostly because the tumor microenvironment is mostly hypoxic and acidic, which creates a suitable environment for spore germination and subsequent growth of the agent [106].

The anticancer activity attributed to bacterial spores is based on their capacity to generate and proliferate in hypoxia and nutrient-rich tumor milieu. The spores, after entering the tumor, develop and grow and release toxic proteins and enzymes that have a direct toxic effect on cancer cells. Presumably, the appearance of these spores in the organism provokes the natural immune response in the host and improves the body’s ability to combat cancer. The spores can also produce secondary metabolisms, which possess anti-tumor properties that increase the effectiveness of this remedy [107]. In addition, these bacterial spores can be genetically manipulated and engineered to incorporate special genes, which make therapeutic proteins that can locate and be expressed within the tumor region, thus increasing the efficiency and effectiveness of anticancer therapy [108].

**Figure 2:**
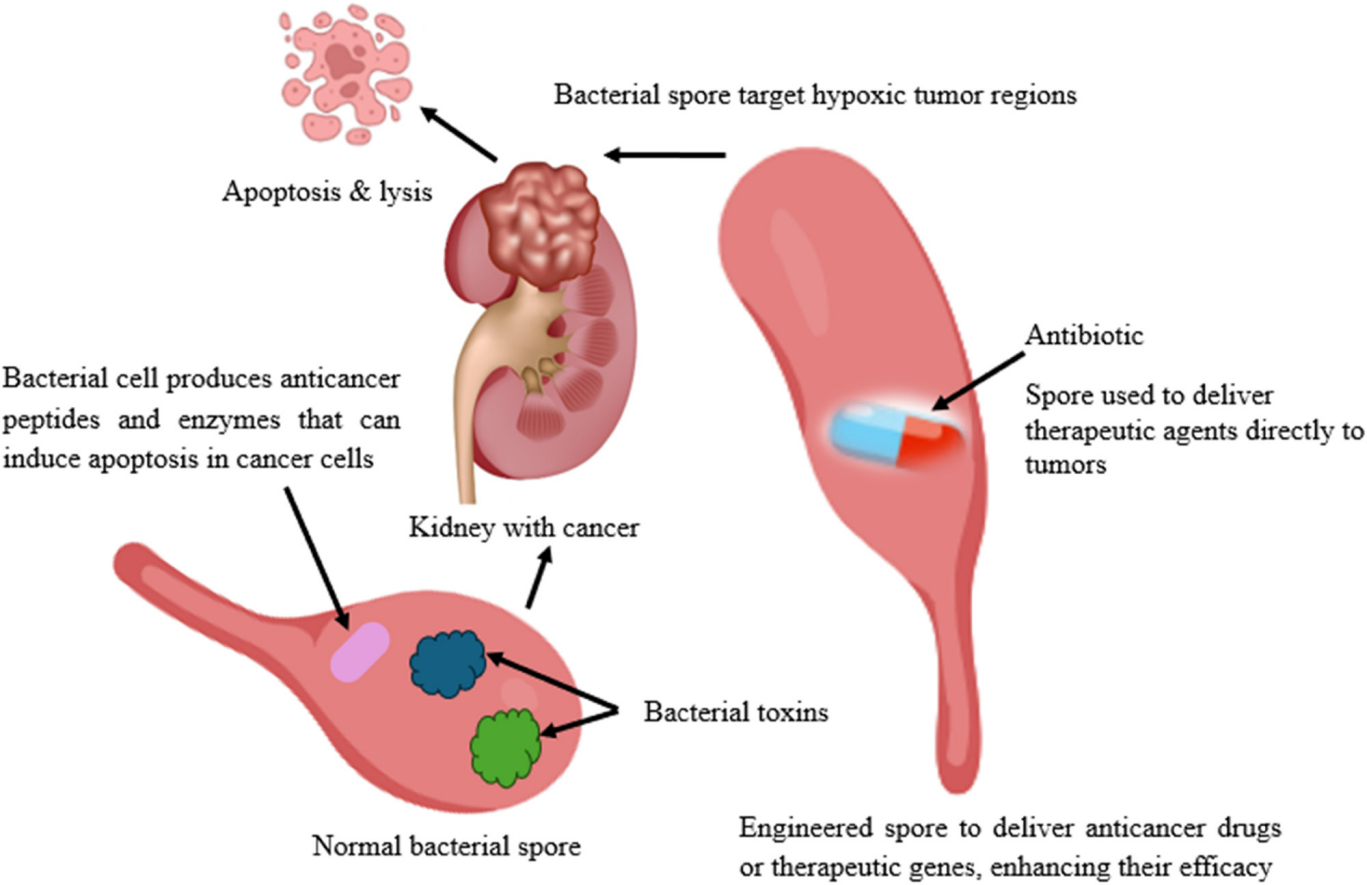
Schematic representation of bacterial spores and their potential as anticancer agents. Created with BioRender.com.

Research has demonstrated that the spores of *B. subtilis* have potential in cancer treatment. It has been reported that the spores of *B. subtilis* could home in on tumor cells and cause regression of tumors. The spores germinate in the hypoxic areas of the tumor, and upon contact with the tumor, the agent releases bioactive compounds, including toxins and enzymes that degrade the matrix and facilitate the invasion of cancer cells, thereby improving therapeutic benefits [109]. Similarly, *C. novyi* -NT is an oncolytic strain of *C. novyi* and is one of the most thoroughly studied strains known so far. *C. novyi*-NT spores selectively germinate only in the hypoxic tumor region and liberate toxins that cause the tumor cells to rupture. This bacterium has been assessed and found to have high anticancer potential in preclinical and clinical trials [110]. *Listeria* spores are able to infect both tumor cells and antigen-presenting cells, resulting in a direct lethal effect on tumor cells and induction of systemic immune response. This strategy has been proven to be effective in preclinical models of a variety of cancers, including pancreatic and ovarian cancers [111]. Figure 2 illustrates the application of bacterial spores as anticancer agents, highlighting their features and applications in cancer therapy. The figure underlines the opportunity to use bacterial spores in cancer therapy based on their specificity and displays the possible applications of such a strategy.

### 4.5 Bacterial toxins and metabolites

Researchers have extensively studied bacterial toxins produced by pathogenic bacteria as potential anticancer agents (Table 3). These toxins, usually proteins or peptides, can disrupt cellular processes, causing cell death. Although bacterial toxins are traditionally known for causing diseases, some have been GM to target cancer cells because of their strong biological effects. This strategy aims to use the natural ability of bacterial toxins to kill cancer cells while minimizing harm to healthy tissues. Studies in this area have led to the development of new cancer treatments that use bacterial toxins in their original form or as part of engineered structures to improve their effectiveness and specificity [112].

**Table 3:** Summary of major bacterial toxins and their metabolites as anticancer agents

Bacterial species/strains	Bacterial toxins and metabolites	Cancer type	Mechanism of action or results	References
*E. coli*	PE38-anti-herceptin and recombinant PE-STXA-anti-herceptin	Breast cancer	Caused apoptosis in HER2-positive breast cancer cells (SKBR-3)	[130]
*Campylobacter jejuni*	*C. jejuni* cytolethal distending toxin B (Cj-CdtB)	Breast cancer	Displayed selective pressure on trastuzumab in combination with HER-2 protein	[131]
*Vibrio vulnificus*	RAS/RAP1-specific endopeptidase (RRSP)	HeLa cell line	Interferes with the RAS signaling pathway	[132]
*Clostridium perfringens*	Enterotoxin (C-CPE) (c-terminal fragment)	Pancreatic and breast cell lines	The anticancer properties of CPE have been generated against claudin-expressing pancreatic and breast cell lines	[133]
*E. coli*	Shiga-like toxin-A (3f7)	Mantle cell lymphoma (CD20-expressing cells)	3f7 demonstrated inhibitory activity against the growth of the cancer cells, partly through apoptotic pathways (mitochondrial apoptotic pathways)	[134]
*Vibrio cholera* (CET40)	Exotoxin of *Vibrio cholera* (CET40)	Burkitt’s lymphoma cell line (Raji), embryonic kidney cell line (293TT), lung adenocarcinoma epithelial cell line (A549), colorectal adenocarcinoma (DLD-1), and human oral epidermoid carcinoma (KB3-1)	The combination of CET40 and human transferrin (HB21) resulted in the proliferation of the cancer cell lines suppressed in all of the cell types, where the most significant effect was observed against the DLD-1 cell line	[135]
*E. coli*	Cytotoxic necrotizing factor (CNF)	Different cancer cell lines	Trigger DNA replication, causing the development of multinucleated cells coupled with suppression of cell differentiation and induced apoptosis	[113]
*pks*-positive *K. pneumonia*	Colibactin	CRC	Colibactin caused DNA damage and inflammation in the course of CRC progression	[136]
*Lactococcus lactis*	Nisin	CRC cell lines	Nistin caused cytotoxic effects against CRC cell lines, such as HT-29 and Caco-2, by lowering the levels of carcinoembryonic antigen linked to metastasis of CRC cells	[137,138]
*K. pneumoniae*	Microcin E492	Colorectal carcinoma cells	Generated cytotoxicity on the HT-29 cell line compared with SW620 by depolarizing the cell membrane potential by pore-forming ability, DNA fragmentation, caspase activity, and phosphatidylserine release. Releasing intracellular calcium ions is the principal mechanism of cellular apoptosis by inducing microcin	[139,140]
*Pediococcus acidilactici* (K2a2–3)	Bacteriocin pediocin	CRC cells such as DLD-1 and HT-29	Cytotoxic effects on CRC cells such as DLD-1 and HT-29	[141]
*Pediococcus acidilactici* K2a2–3	Pediocin K2a2–3	HT-29 colon adenocarcinoma cells	Cytotoxic effect against HT-29 colon adenocarcinoma cells because of the hydrophobicity of this peptide	[141]
*Enterococcus* sp.	Enterococcal anti-proliferative peptide (Entap)	Mammary gland adenocarcinoma (MDA-MB-231), prostatic carcinoma (22Rv1), uterine cervix adenocarcinoma (HeLa), colorectal adenocarcinoma (HT-29), and human gastric adenocarcinoma (AGS) cell lines	Arrest cancer cells in G1 and stimulate autophagic apoptosis	[138]
Marine bacterium *Bacillus sp.*	Mixirins	Human colon tumor cell line	Block the proliferation of HCT-116	[142]
*Mechercharimyces asporophorigenens* (marine actinobacterium)	Urukthapelstatin	MCF-7, lung cancer (NCI-H460, A549, and DMS-114), ovarian cancer (SK-OV3, OVCAR-3, OVCAR-5, OVCAR-8, and OVCAR-4), and colon cancer (HCT-116)	Exhibits anticancer activity by inhibiting the growth of all cancer cell lines tested	[143]

The role of microorganisms in combating cancer was first observed about 150 years ago following the oncolytic effect of erysipelas *S. pyogenes* infections observed in hospitalized cancer patients. Also, in the mid-nineteenth century, it was reported that regression of tumors was noted in patients experiencing skin infection by *Streptococcal* bacteria. These discoveries facilitated the development of cancer immunotherapy and the use of a vaccine for killed bacterial species, referred to as Coley’s toxins. Coley’s toxins were also found to be useful in delaying the regression of several terminal malignancies, such as myelomas, carcinomas, lymphomas, melanomas, and sarcomas. This pioneering work laid the groundwork for what became the potentially promising area associated with microbial toxin-induced cancer therapy [113].

The efficacy of bacterial toxins as anticancer agents is largely attributed to their capacity to impair crucial cellular activities in cancer cells. For example, one mechanism is the inhibition of protein translation. Diphtheria toxin (DT) produced by *Corynebacterium diphtheriae* inactivates elongation factor-2, thus inhibiting protein translation and leading to cell death [114]. Similarly, exotoxin A, produced by *P. aeruginosa*, inhibits protein translation through adenosine diphosphate-ribosylation of elongation factor-2 [115]. Other toxins, such as *Clostridium perfringens* enterotoxin, target a specific cell receptor and affect cytoplasmic membrane integrity, resulting in cell lysis [116]. Also, toxins can trigger apoptosis, such as anthrax toxin produced by *Bacillus anthracis,* by impairing intracellular signaling pathways. Collectively, these represent the multitude of mechanistic strategies bacterial toxins leverage to induce cytotoxicity in cancer cells. Given this, bacterial toxins represent an attractive modality in cancer treatment [37].

The potential benefits of using bacterial toxins as a treatment for cancer are noteworthy for many reasons. A major benefit is potency; bacterial toxins are often effective in low amounts, meaning that systemic toxicity is negligible [117]. Another benefit is that scientists could enhance the specificity of cancer cells using genetic engineering to produce fusion proteins integrating toxins with specificity, often in the form of antibodies or growth factors. This strategy would prevent unintended damage to normal tissues. For example, immunotoxins (antibodies fused with toxins) have shown promising efficacy for a range of cancers, hematological malignancies, and solid tumors in preclinical studies and could provide a pathway forward to market [118]. Additionally bacterial toxins could bypass resistance. Because bacterial toxins often engage a different cellular pathway than traditional chemotherapeutics, bacterial toxins present an opportunity for drug-resistant cancer cells [119]. In general, based on their unique mechanisms and advances in biotechnology, bacterial toxins have the ability to be both effective and versatile anticancer agents.

There are certain merits of employing bacterial toxins as anticancer agents. One of the advantages is that bacterial toxins are naturally potent biological compounds capable of inducing the death of targeted cells at low concentrations, thereby minimizing toxicity to surrounding cells. In addition, the selectivity of the toxin to the cancer cells can be further improved by GE for the development of hybrid proteins in which toxins are conjugated with targeting ligands such as antibodies or growth factors. Another advantage is the capability to counter drug resistance because bacterial toxins act through pathways that differ from those of chemotherapeutic agents [119]. In general, due to specific characteristics of bacterial toxins, as well as the rapid paradigm shift in the GE, they are promising frontiers for the emergency of effective anticancer drugs.

DT, derived from the bacterium *C. diphtheriae*, was chosen as the basis for the development of the first immunotoxin, denileukin diftitox (ONTAK), due to its potent cytotoxic properties and convincingly well-understood mechanisms of action. As a protein synthesis inhibitor, DT acts by ADP-ribosylating elongation factor-2, which interrupts cellular function and leads to cellular death. In denileukin diftitox, the bacterium’s toxin was also modified to contain interleukin-2 (IL-2), which targets the IL-2 receptor on the surface of IL-2-expressing cells, such as certain cancer cells. It is the strategic and functional targeting of DT that enhances the specificity of the action of DT to selectively kill cancer cells and spare normal cells. Denileukin diftitox has been especially effective in the treatment of cutaneous T-cell lymphoma with notable clinical efficacy, leading to its approval for therapeutic use [119].

*C. perfringens* enterotoxin (CPE) has displayed frontier anticancer properties, predominantly in cancers that developed from epithelial cells, including ovarian and CRC. CPE can target two important proteins, i.e., claudin-3 and -4 (tight junction proteins), which are commonly expressed in cancer cells. Research findings revealed that when CPE was introduced in an ovarian cancer animal model, it resulted in the binding of CPE to claudins and provoked rapid cytolysis and cell death. The binding of CPE to claudin-4 in CRC cells also demonstrates cytotoxicity by causing the immediate loss of tight junctions along with cell death. Altogether, researchers suggest that CPE can exploit the differential expression of epithelial-derived claudins in cancer cells and selectively induce cell death [120].

*P. aeruginosa* exotoxin A (PE) has shown very unique anticancer properties by interfering with the process of protein synthesis in cancerous cells. For instance, PE has been conjugated with other targeting ligands to synthesize PE-based immunotoxins, such as PE38 immunotoxin, an immunotoxin consisting of an antibody linked to PE38 that targets cancer cells. The SS1P immunotoxin, which consists of PE38 linked to an anti-mesothelin antibody, has shown effectiveness in treating mesothelioma and other tumors, which overexpress mesothelin in preclinical as well as clinical trials [121].

*Staphylococcus aureus* enterotoxin B (SEB), for instance, has also been identified to possess anticancer activity. This superantigen activates T cells and generates cytokines and enhances anti-tumor immunity. In other studies, it was found that SEB could enhance immune responses against tumor cells using murine models like melanoma and lymphoma. As potent as this toxin is in producing an immune response, it may be administered in cancer immunotherapy. Considering how powerfully this toxin induces an immune response, it could be used in cancer immunotherapy [122–125].

*P. aeruginosa* exoenzyme S (ExoS) is the other notable example of a multifunctional toxin, which is known for its anticancer activity, and acts by disrupting cell signaling pathways involved in cancer progression. According to documented reports, ExoS can downregulate the activity of Ras and Rho GTPases, which are significant regulators of proliferation and survival of cells, which ultimately induce apoptosis and inhibit the growth of cancer cells as observed in various models, including melanoma and lung cancer [126].

*Bacillus anthracis* is known to synthesize the anthrax lethal toxin (LT), and this toxin could have noteworthy anticancer properties. The LT of anthrax is composed of lethal factor (LF) and protective antigen (PA). LF works as a zinc-dependent metalloprotease that destroys the target proteins belonging to the MAPKK family by cleaving them in the cytosolic part of the host cell after its penetration inside the body. By interfering with the MAPK signaling pathways, the cancer cells lose their ability to survive, and apoptosis is triggered. The LT of anthrax has also been reported to have the ability to target the cancer cells selectively and spare the normal tissues, as observed in preclinical models [127].

Recently, scholars have identified that the secondary metabolites (lipopeptides) in *B. subtilis* strain Z15 (BS-Z15 SMs) exhibit an inhibitory effect on cancer cells, particularly liver cancer cells (BEL-7404). These metabolites trigger apoptosis and cause G0/G1 phase arrest, resulting in a decline in the potential of the mitochondrial membrane. The overall mechanism of action suggests that apoptosis specifically occurs through the mitochondrial pathway [128].

Niamah et al. reviewed the anticancer activity of bacteriocins derived from lactic acid bacteria. The review explores the mechanisms through which these bacteriocins operate. It also demonstrates the potential technical advancements that could maximize the efficacy of bacteriocins in the treatment of different types of cancers. In addition, the authors also displayed some of the challenges that need to be mitigated for better and effective utilization in clinical settings [129].

### 4.6 Bacterial biofilms

Over the years, bacterial biofilms, which cause chronic infections and are linked to medical implant-related complications, have been identified as a possible modality of cancer treatment. Biofilms are complex microbial communities formed with bacteria, and they are enclosed by a protective layer known as an extracellular matrix that hinders them from the host’s immune system or antimicrobial agents. This endurance, along with their capacity to keep on to the surface and build intricate form, has urged interest in using biofilms for targeted delivery of therapeutic agents to carcinoma cells [144].

The utility of bacterial biofilms in cancer therapy is rooted in their capacity to access and infiltrate tumors readily. Some biofilms can be engineered or naturally selected that may selectively target TME because these sites are hypoxic and acidic in nature and are common features of solid tumors [145]. This selective targeting can improve the biodistribution of therapeutic agents, including chemotherapeutic drugs or nanoparticles, to cancer cells while reducing systemic exposure, thus minimizing off-target toxicity. Additionally, encapsulation of agents within the biofilm matrix enhances their stability by protecting them from degradation by enzymes or other factors, hence enhancing the release of bioactive molecules into the TME over an extended period [145].

The development of biofilm by bacterial pathogens on cancer cells triggers metastasis disruption. Hence, bacterial biofilm demonstrates feasible applicability in the treatment of cancer [146]. It has also been realized that anticancer therapeutic agents can induce the development of biofilm during therapeutic intervention, which causes the distraction of metastasis [147]. Research findings indicated that bacterial biofilm can impact the progression and development of colon cancer via the modification of cancer metabolome to generate a regulator of cellular proliferation [146]. Besides, the bacterial macromolecules essential for the formation of biofilm, such as DNA and proteins, shield cancer cells to stop metastasis [148]. For instance, polysaccharides produced by *Streptococcus agalactiae* hinder the adherence of cancer cells to endothelial cells, a crucial stage in cancer metastasis [148]. In a different investigation, Kumeria et al. [149] display the prospective use of iron oxide nanowires extracted from biofilm byproducts of the bacteria *Mariprofundus ferroxydans* as a new multifaceted drug vehicle for the treatment of cancer and cancer hyperthermia. In alternative research, biofilm developed by *Lactobacillus reuteri* was utilized to generate nanoparticles (mesoporous silica) for the directed delivery of 5-FU in the colorectum, and this biofilm, when covered with zinc gallogermanate promoted CRC imaging [150].

### 4.7 Bacterial proteins and enzymes in prodrug therapy

Various bacterial proteins may inhibit cancer through various mechanistic avenues. One of the notable bacterial proteins that demonstrated oncolytic activity against cancer cells is Azurin. Azurin is a bacterial protein metabolically produced from *P. aeruginosa*. Azurin facilitates the apoptotic process in neoplastic cells through the stabilization of the tumor suppressor protein p53, which results in cell cycle arrest and subsequent cell death [151]. *Mycobacterium bovis* also synthesized very crucial protein with endostatin-like effects, which exert an inhibitory effect for angiogenesis and metastasis [152]. Moreover, ActA is a special protein secreted by *L. monocytogenes* that can elicit a more prominent immune response to elicit greater recognition and cellular attack toward neoplastic cells [153].

**Figure 3:**
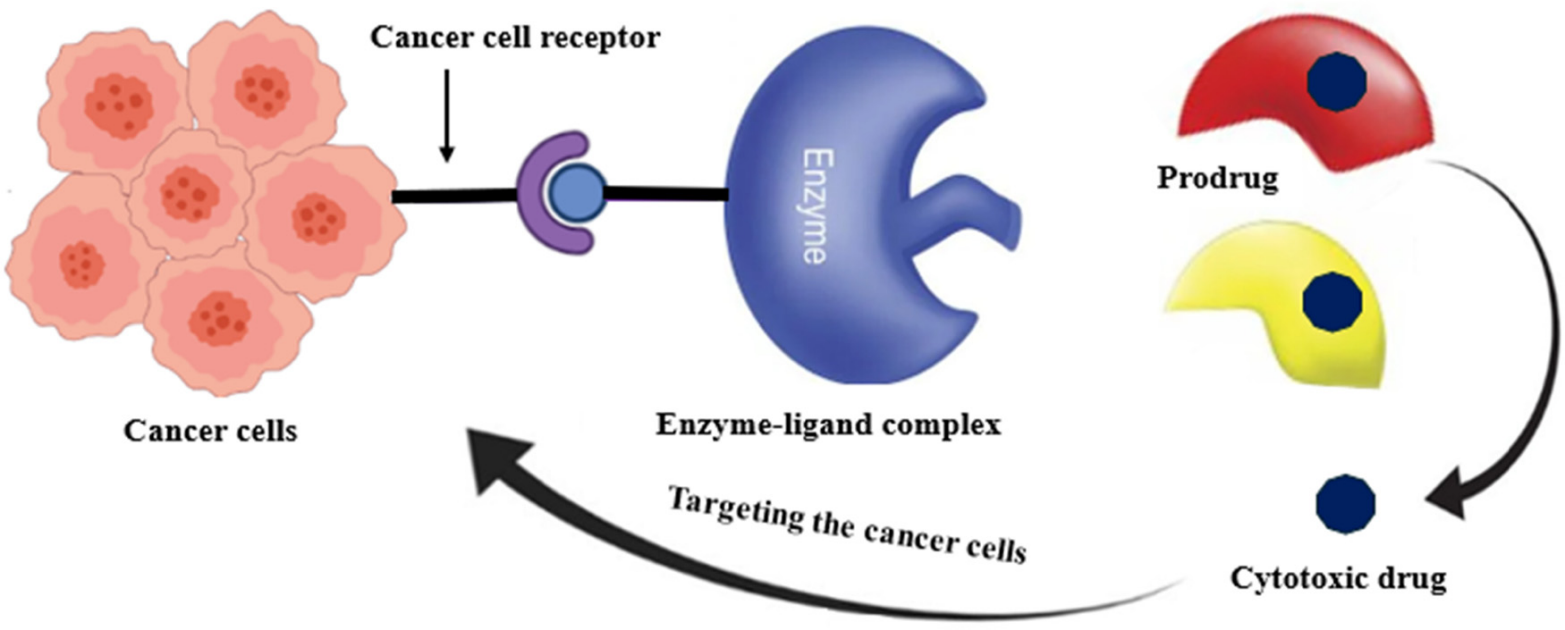
Schematic illustration of the mechanism of bacterial enzyme prodrug cancer therapy. Created with BioRender.com.

Bacterial enzymes play a crucial role in prodrug therapy, especially toward cancerous cells. This strategy involves using an inactive compound that is toxic only when given as a drug and accumulates at the tumor site without affecting the rest of the body. The process begins with systemic delivery of the prodrug after which bacterial enzymes are localized to the tumor site either via vectors (bacterial) or as purified enzymes. These enzymes then activate the prodrug within the TME, thus leading to the selective killing of tumor cells without affecting normal cells (Figure 3) [154,155].

Bacterial enzyme prodrug therapy offers several advantages. First, bacterial enzymes are very selective, so the prodrugs are activated only in the tumor region, which in turn minimizes the side effects. Second, the prodrugs are inactive and only become active when they reach the tumor sites; therefore, patients treated with prodrug-based chemotherapy suffer fewer side effects compared to those who undergo usual chemotherapy [156]. Besides, bacterial enzyme–prodrug systems can be employed in synergistic therapy with other treatments, such as immunotherapy and radiotherapy, to boost the efficiency of total treatment [157]. Nevertheless, bacterial enzyme prodrug therapy has several limitations and directions for future research. One limitation is the ability to deliver the bacterial enzymes to the tumor site and safely distribute them while avoiding harm to the body; researchers are currently exploring nanotechnology and other delivery systems to address this issue. Furthermore, the human body’s immune system may eliminate bacterial enzymes or vectors before reaching the tumor, and, therefore, strategies to alter immune response become mandatory. In addition, although there are increasing numbers of preclinical studies, more research is needed in this area to assess the safety and effectiveness of these therapies in clinical trials before such therapies can be applied in the clinical setting [158,159].

Cytosine deaminase (CD) is an enzyme that biotransforms the non-toxic prodrug 5-fluorocytosine (5-FC) into the toxic chemotherapeutic compound 5-fluorouracil (5-FU). This mechanism has proved useful in cancer treatment since the transformation of 5-FC to 5-FU is local in the case of malignant cells, resulting in the death of the cancerous cells with minimal side effects to the rest of the body. This approach has been implemented in different cancer models and has proved to have a huge potential to be used to treat cancers such as CRC as well as gliomas. Carboxypeptidase G2 (CPG2) is another enzyme that activates nitrogen mustard prodrugs by converting them into their cytotoxic forms. This enzyme–prodrug system is promising in the treatment of solid tumors, and further research focuses on increasing the targeting ability and the advantages of the enzyme and prodrug system. Research has been conducted to analyze different approaches to enhance the targeting efficacy of CPG2 so as to improve the disease benefits without effects on normal tissues [160]. Likewise, beta-glucuronidase is an enzyme that cleaves the glucuronide prodrugs to liberate active chemotherapeutic agents. This system is especially effective in tumors that express high levels of beta-glucuronidase, which includes some types of breast and colon cancers. The localized presence of the enzyme beta-glucuronidase within the TME enables the selective activation of the prodrugs, thus increasing the selectivity and decreasing the systemic toxicity of the treatment [161].

## 5 GE and naturally occurring oncolytic bacteria

**Figure 4:**
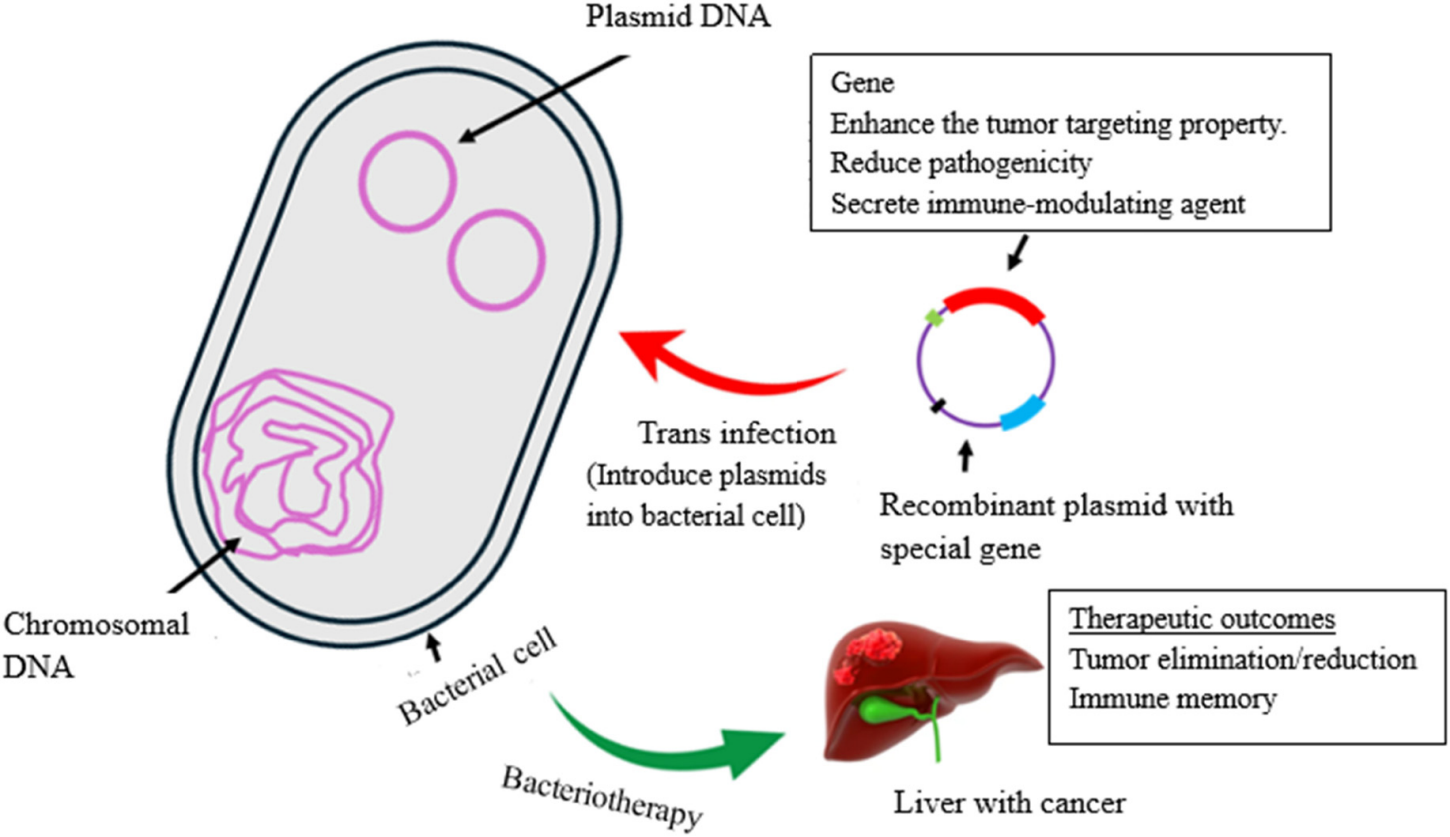
Schematic diagram showing the link between genetic modification and oncolytic therapy. Created and modified with BioRender.com.

Genomic engineering is the process of manipulating bacterial genomic sequences by employing genetic engineering tools (Figure 4). These GE bacteria have the ability to synthesize altered proteins, nucleic acids, peptides, and other biomolecules that are useful in treating various diseases. They can selectively target and destroy affected tissues or organs, identify specific biomarkers of diseased areas, and alter the colonized area to enhance the effectiveness of disease treatment (like breaking down physical barriers to improve the delivery of drugs) [162].

Oncolytic bacteria are developed through GE aimed at increasing the specificity of the bacteria to tumor tissues and improving the therapeutic efficacy. In the process of genetic engineering, first the target bacterium should be isolated from the naturally occurring environment and alter their DNA to attain a particular therapeutic goal. This process includes the addition or removal of specific genes that regulate different functions of a bacterium, including movement, the ability to avoid being destroyed by the host immune system, and the ability to produce toxins, among others [162].

One of the major benefits of GE oncolytic bacteria is their specificity and potency toward the targeted cancer cell [163]. For example, they can be developed to generate therapeutic proteins or antigens that trigger the immune response to have more potency to destroy cancerous cells. Moreover, these bacteria can be designed to contain genetic circuits, which trigger the response to the signals found exclusively in the TME, thus improving their selectivity and the ability to deliver therapy to the cancer cells while leaving the normal cells untouched [164].

However, the success of GE-based cancer therapy does not only rely on the efficacy of the therapeutic approach but also on the safety of the preparation is very crucial to maintaining its use for humans [165]. Regulatory approval activity comprises several important safety aspects, including the natural behavior of the agent, toxicity levels, and potential side effects, which are achieved via preclinical assessment. Despite these activities, ongoing progress in GE techniques continues to broaden the therapeutic potential of GE-based oncolytic bacteria, generating promising roadmaps for personalized cancer therapy.

### 5.1 Salmonella typhimurium

*Salmonella enterica* serovar typhimurium (*S. typhimurium*) is a facultative anaerobic bacterium that is well-documented to possess potential anti-tumor properties. It shows direct cytotoxicity on tumors and is used for the selective administration of anticancer agents [29].

According to a report by Hayashi et al., a GM strain of *S. typhimurium*, termed A1-R, has been developed as a targeted therapy for cancer metastasis. This strain requires both arginine and leucine for growth and is capable of growing in cancer cells but not in the normal cells of the body. When injected into the lymph nodes and the bloodstream in mouse models of pancreatic cancer and fibrosarcoma, A1-R selectively localized and killed metastatic tumors over the course of 7–21 days, as visualized by real-time imaging. This approach appears quite effective in managing metastatic cancers and is completely safe without the use of chemotherapy [166].

Tan et al. investigated the anticancer activity of GM *S. typhimurium* against both stromal cells and cancer cells in pancreatic tumors. In this study, cytolysin A (ClyA) produced GM *S. typhimurium* was introduced into nude mice with either subcutaneous or orthotopic human pancreatic cancers. Following intravenous administration of the bacterium, researchers examined the markers on stromal cells, changes in the tumor surroundings, stromal cell counts, and the infiltration of immune cells. Results obtained from this experiment revealed that the GM strain selectively aggregated and grew within tumor tissues. The retarded growth in pancreatic cancer is attributed to the ClyA produced by the agent. This approach suggested the ability of the bacterium to penetrate tumor-linked stromal barriers and exhibit anticancer effects [74].

A study by Zheng and Min [167] explored the novel ability of an attenuated strain of *S. typhimurium* strain ΔppGpp (guanosine 5’-diphosphate-3’-diphosphate) (strains deficient in guanosine pentaphosphate and tetraphosphate) to selectively target and perpetuate within cancer-harboring tissues, resulting in a considerable reduction in the size of the tumor or being fully eliminated in the mice model. The study displays the role of ΔppGpp *S. typhimurium* strains as vectors for introducing therapeutic payloads selectively to tumors.

Recently, Sun et al. studied the GE oncolytic bacterium HCS1, comparing its activity to the attenuated *S. typhimurium* strain VNP20009. *In vitro* laboratory analysis showed that HCS1 had significantly lower colonization and cytotoxicity against cancer cells compared to VNP20009. The administration of HCS1 to tumor-bearing mice resulted in considerable suppression of cancer growth in a dose-dependent manner without causing adverse effects in healthy tissue, suggesting its potential in cancer treatment with an improved safety profile [168].

### 5.2 Listeria monocytogenes

Current and past investigations on GE *L. monocytogenes* demonstrate the inhibitory activity of this bacterium against various types of cancers, highlighting its potential in oncolytic therapy.

According to Vitiello et al., attenuated *L. monocytogenes* (Lmat-LLO) serve as a potential anticancer agent and delivery strategy for the treatment of different types of cancers. *In vitro* studies show that Lmat-LLO causes ROS generation and apoptotic cell death in numerous melanoma cell types, independent of stage, mutational profile, susceptibility to BRAF inhibitors, or stemness. In Braf/Pten genetically altered mice, Lmat-LLO lowers the size and volume of initial melanoma tumors while also decreasing metastatic spread [169].

Jia et al. developed a GE *L. monocytogenes* strain that expresses the HPV16 E7 protein. Vaccinating mice with cervical cancer intraperitoneally with the modified strain (LM4Δhly: E7) resulted in considerable tumor size reduction and even total remission of certain existing tumors. The study demonstrated that the recombinant *L. monocytogenes* may enter tumor tissues and promote non-specific tumor cell death, most likely via the production of ROS and raised intracellular levels of calcium ions [170].

Currently, a group of researchers indicated that *L. monocytogenes* mutated for oncolytic use has shown inhibitory activity toward different types of cancers. These modified bacteria are designed to selectively infect and replicate within tumor cells without affecting healthy cells. In the process of oncolytic activity caused by *L. monocytogenes*, the GE *L. monocytogenes* selectively infect tumor cells because of their capability to thrive in the hypoxic environment typical of solid tumors. Once internalized into the tumor cells, the agent induces lysis of the targeted cell through the potent toxins released for the bacterial cell [171]. Besides, GE *Listeria* can be organized to express tumor-linked antigens, improving the ability of the immune system to recognize and fight the cancer cells.

### 5.3 E. coli

Chiang and colleagues used the probiotic *E. coli* Nissle 1917 (EcN) strain for the treatment of CRC. In this study, the cytotoxic protein HlyE, which is regulated by the araBAD promoter (PBAD), was engineered into EcN. EcN was metabolically engineered to decouple glucose-mediated control and l-arabinose transport induction while simultaneously blocking l-arabinose catabolism in order to overcome the drawback that PBAD was sensitive to glucose and prone to all-or-no induction. EcNe, the reprogrammed strain, allowed for the effective and time-controlled expression of HlyE. At 30- to 50 μM l-arabinose concentrations, EcNes HlyE production reached its peak and was not impacted by glucose. In mice xenografted with human CRC cells, the engineered EcNe markedly decreased tumor size and specifically colonized tumors with a tumor-to-organ ratio of 106:1. This study introduced a novel approach for bacteria-mediated delivery of therapeutic proteins to tumors [172].

Apart from GE oncolytic bacterial species, naturally existing oncolytic bacteria strains have been reported for their unique capability to specifically attack and eradicate cancer cells. For instance, the two commonly occurring bacterial strains, such as *C. novyi* and *S. pyogenes,* flourish tumors cell and eliminate cancer cells without causing significant damage to normal tissues [163,173].

The most prominent benefits and successes of oncolytic therapy linked to naturally occurring oncolytic bacteria lie in their evolutionary adaptation to TMEs, where this therapeutic frontier gains an advantage over GE counterparts. The proliferation and survival of this agent within the tumor cell are partly associated with the natural selection processes, which can boost their therapeutic potential and minimize the possibility of resistance occurring in cancer cells [139].

In spite of the peculiar oncolytic characteristics of naturally occurring bacteria, these bacteria may necessitate some form of changes or improvements to enhance their therapeutic uses for clinical cases. The combination of naturally occurring oncolytic bacteria with immunotherapies or other cancer treatments is being studied to enhance their specificity, unlocking their mechanism of action and overall therapeutic outcomes [139].

### 5.4 Clostridium spp.

*Clostridium* species are known for their unique oncolytic activity as the agent targets the cancerous cells and selectively colonizes the tumor cell. This specificity is guaranteed because the hypoxic and necrotic areas, which are characteristic only for solid tumors, are suitable for the germination of *Clostridium* spores, thereby killing the tumor cells while having little effect on the normal cells [174]. This tumor-selective colonization not only assists in the direct destruction of tumor cells through bacterial lysis but also enables the targeted deposition of therapeutic agents synthesized by the bacteria. Such kind of approach is also very helpful in reducing the side effects often associated with conventional treatments [175].

The anticancer activity of *Clostridium* species and its spore, mainly *C. novyi*-NT, was reviewed by Staedtke et al. and Feng et al. [176,177].

### 5.5 Bacillus spp.

*Bacillus* species, such as *B. subtilis* and *B. thuringiensis,* have been recently explored for their oncolytic properties in cancer treatment [178,179]. These bacteria have inherent properties that are beneficial for use in targeted cancer therapy. Moreover, their capacity to dwell in hypoxic TMEs and their specificity to eliminate cancer cells and not harm normal cells further emphasize their therapeutic utility. Furthermore, *Bacillus* species can be GM to increase their ability to kill cancer cells and demonstrate a therapeutic load containing anticancer drugs or immunomodulators directly to cancer tissues. Current research remains focused on elucidating the molecular pathways through which they exert their oncolytic effect and the optimization of their use in clinical practice [90,180].

Recently, Zhao et al. assessed the anticancer potential of four bacterial strains (BY38, BY40, BY43, and BY45), which were isolated from the fecal samples of healthy persons as well as cancer patients. According to the results obtained from this study, the tested organisms significantly suppress the growth of ovarian and CRC cell lines in a dose-dependent manner, suggesting the possibility of these products being used as alternative therapeutic agents for these forms of cancer [181].

### 5.6 Bifidobacterium

Facultative anaerobes like *Bifidobacterium* are bacteria that cannot survive in an environment when the level of oxygen is high. This characteristic forces them to move toward the hypoxic areas inside the tumors where oxygen is scarce [182].

Recently, a group of researchers investigated the effects of heat-killed *Bifidobacterium* and *Lactobacillus* strains on the MKN1 human gastric carcinoma cells using animal models and *in vitro* assays. In this study, the MKN1 xenograft models were established by implanting BALB/c nude mice with MKN1 cells and then the mice receiving the tested bacterial strains orally as single or in combination. Western blotting and immunohistochemistry of the tumor tissues showed that the strains *L. reuteri* MG5346, *B. bifidum* MG731, and *L. rhamnosus* MG5200 were capable of eliciting greater apoptosis in MKN1 cells compared to other strains. Besides, these strains, especially MG731, showed a significant reduction in the growth of tumors in these animal models [183].

According to the study by Faghfoori et al., *Bifidobacteria* had anticancer properties on colon cancer cell lines, and the findings showed that it was able to significantly decrease the survival rate of the colon cancer cells compared to that of the control groups. The data obtained by flow cytometry and reverse transcription polymerase chain reaction proved that metabolites produced by *Bifidobacteria* led to apoptosis in colon cancer cells. According to the authors, the effect was attained by the suppression of anti-apoptotic genes along with the promotion of pro-apoptotic genes [184].

In a recent study published by Yoon et al., the administration of two different strains of *B. breve* suppressed the proliferation of tumors in mice with MC38 colon carcinoma. The results revealed that only one of these strains improved the performance of the chemotherapeutic agent used (oxaliplatin and PD-1 inhibitors). According to the immune profiling and transcriptomic analysis, this strain of *B. breve* increased the anticancer immune response by stimulating lymphocytes. The authors concluded that the combined effect of *B. breve* and the tested antibiotics could be a viable strategy to harness the potential of *B. breve* to enhance the effectiveness of CRC treatments [185].

Bahmani et al. isolated 17 isolates of *Bifidobacterium* from diverse sources and tested them against colon cancer. According to the findings of the study, *Bifidobacterium* was detected frequently in infant fecal samples and dairy products, with the least prevalence in local milk. In this study, both the infant feces-derived isolate (cell-free supernatant (CFS) and one from the probiotic capsule showed maximum inhibition of cancer cell growth on a colon cancer cell line. Out of all the probiotics, *B. bifidum* was especially effective against cancer cells and received a vast improvement in the results of gastrointestinal cancer. In conclusion, this study stated that the CFS produced by these isolates can suppress the growth of colon cancer cells; thus, it shows that probiotics have high potential as a new therapeutic strategy for colon cancer [186].

In a recent study, Fahmy and co-workers explored the modulatory impact of *B. longum* (BL), which was isolated from women’s breast milk samples, on selected oncomiR and tumor-suppressor miRNA, as well as on IL-1β and IL-6-targeted miRNA, employing the mice model of inflammation-induced CRC. The study observed that BL administration inhibited miR-21a and miR-155 oncomiRs that modulated IL-6 and IL-1β in both normal and CRC mice. It also upregulated tumor suppressor miRNAs, including miR-145 and miR-15a of the tumor cells. Furthermore, BL treatment downregulated the expression of miR-146a, which regulated the expression of IL-1β and IL-6. In CRC mice, the raised concentration of NF-Kb was, however, reduced by BL. Alternatively, in CRC mice, BL caused an increase of IL-1β but a decrease of IL-6. In addition, reduced levels of aberrant crypt foci in CRC mice were observed under the BL treatment, while increased necrosis and fibrosis in colon cells were also observed. The results obtained in this study suggest that BL modulation of microRNAs could provide positive therapeutic outcomes in CRC by inhibiting tumor cell proliferation, apoptosis, and related activities [187].

## 6 Safety and side effects

The application of bacterial enzymes, toxins, spores, and GE bacteria for the treatment of cancer is a novel approach but has some safety concerns. Oncolytic bacteria-linked therapies are primarily introduced to exploit the inherent nature of the bacteria to be toxic to cancer cells without harming the intact cells. However, safety concerns are mainly centered on the prospect of systematic infection, immunogenicity issues, and off-target effects. Of all, cytotoxicity is a potential hazard associated with bacterial toxins and spores. Despite the attempts to deliver these agents selectively to the tumor site, there is a possibility of unintended damage to the normal tissues. For instance, *C. novyi*-NT spores that possess oncolytic activity can lead to septicemia if it is not efficiently delivered within the TME [110]. Likewise, bacterial toxins, such as anthrax LT, exhibit a toxicity profile across the body since they affect systemic organs, causing severe reactions in patients if not well controlled.

GE bacteria suffered from safety concerns linked to their stability and unintended genetic changes, leading to potential treatment failures. While these agents are developed to maximize the potency and specificity of tumors, there is a risk of unintended consequences introduced to the recipient, which may aggravate the disease. For instance, GE *S. typhimurium*-linked oncolytic therapies have shown promising frontier in destroying different types of cancers but worries about the reversion to virulence or unintended infection in immunodeficient individuals remain. Hence, preclinical and clinical testing are strictly required to investigate these risks and ensure patient safety [188,189].

## 7 Combination therapy

Traditional cancer treatments have long been based on approaches such as radiotherapy, chemotherapy, and immunotherapy, which are considered the baseline of cancer management. Nevertheless, these conventional strategies often come with significant drawbacks, including systemic toxicity, drug resistance, and immune suppression, which can adversely affect the health of cancer patients. In this context, the integration of bacteriotherapy with conventional cancer therapies has emerged as a promising strategy [190].

Given that cancer is a multifaceted disease, the approach of employing a combination of internal and external attack methods in targeting both hypoxic and oxygen-rich cells is regarded as a desirable and potentially advantageous option against cancer treatment [191].

Combination therapy of chemotherapies and oncolytic bacteria demonstrated efficacy in early *C. novyi*-NT investigation of a therapy termed COBALT or combination bacteriolytic therapy [191]. *Salmonella* shows potential to boost the efficacy and safety of chemotherapeutics such as cyclophosphamide [192], cisplatin [193], doxorubicin [194], gemcitabine [195], and combinations thereof (e.g., CHOP [196]). Radiotherapy linked to oncolytic bacteria therapy has limitations because of harmful effects on normal tissues with increasing dose and frequency. While there is evidence of synergistic effects for the combination of oncolytic bacteria and radiation [197], the greater share of benefits demonstrated is thought to be because of selective colonization and immunomodulatory effects [198]; however, combinatorial therapeutic approach resulted in superior efficacy relative to systemic administration alone [191,199,200]. Table 4 provides a summary of the anticancer effects generated from the combined use of oncolytic bacteria with commonly used chemotherapeutic agents.

**Table 4:** Summary of studies on the combination of conventional therapeutic approaches and oncolytic bacteria

Bacterial species/strains	Conventional therapy	Cancer type	Outcomes	References
*S. typhimurium* ∆ppGpp	Radiotherapy	Colon cancer	Reduces the growth of tumors in comparison to bacterial therapy alone	[201]
Lipid A mutant *S*. *typhimurium*	X-rays	B16F10 or Cloudman S91 melanoma	Enhances combined efficacy in B16F10 or Cloudman S91 melanoma-bearing mice	[202]
Spores of *C. novyi-*NT	Radiation therapy (external beam radiation)	HCT116 tumors	Reduction in tumor size in mice-bearing HCT116 tumors. Allow sick individuals to be treated with a minimized dosage of radiolabeled antibodies, consequently sparing normal tissues from damage	[198]
*C. novyi*-NT spores	Dolastatin-10, cyclophosphamide, mitomycin C, and vincristine	B16 melanoma and HCT116 colon cancer cells	Massive hemorrhagic necrosis of tumors often occurs within 24 h and has– demonstrated remarkable and long-term anti-tumor effects	[191]
*C. novyi*-NT spores	Microtubule-adhering agents (combretastatin a-4 prodrug and D10)	CRC	D10 considerably boosts the ability of *C. novyi*-NT spores to lyse tumors, while the addition of the tumor cell agent, CTX, further improves the efficacy of COBALT	[203]
*C. novyi*-NT spores	Discodermolide analogues (microtubule stabilizers)	HCT116 tumor	Causes complete and rapid regression of tumor in HCT116 tumor-bearing mice. Enhances the germination of *C. novyi*-NT spores in the hypoxic compartment by widening the area, causing quick regression of tumors	[204,205]
*C. novyi*-NT	Liposomal doxorubicin	Glioblastoma	Fully eliminates tumors, substantially enhances the rate of tumor clearance in glioblastomas in rats	[206,207]
*S. typhimurium* A1-R	Gemcitabine	Pancreatic cancer PDOX	Maximizes the anti-tumor potential of gemcitabine	[195]
Attenuated *S*. *typhimurium* strain VNP20009	Metronomic cyclophosphamide	B16F10 melanoma	Enhances the outcome of the conventional maximum safe dose and minimal dose	[192,208]
Attenuated *S. typhimurium* strain VNP20009	Triptolide	Melanoma	Improved anti-tumor potential	[208])
*S*. *typhimurium* DSLpNG	Doxorubicin	Breast cancer	Inhibits tumor growth rate more efficiently than low-dose doxorubicin alone or *Salmonella* alone. No clinically relevant adverse effects are documented (maximize Treg and CD8^+^ T cells infiltration in the tumors)	[194]
*S. choleraesuis*	Cisplatin	Hepatoma (murine ML-1 cell line); lung tumor (murine LL/2 cell line)	Minimizes the growth of tumors in both lung tumors and slowly growing hepatoma models. Longer survival span (an increase in the number of infiltrating neutrophils, CD8^+^ T cells, and apoptotic cells is displayed in tumor tissues)	[193]
*S. typhimurium* LVR01	A combination of 4 therapeutic agents chemotherapy (doxorubicin, vincristine, cyclophosphamide, and steroid/prednisone)	B-cell non-Hodgkin lymphoma (murine A20 cell line)	Slows tumor progression in comparison to individual therapy by upregulating the expression of Cxcl1 gene. Enhanced the overall health status of mice	[196]

## 8 Clinical trials of oncolytic bacteria

In 1891, Dr. William B. Coley introduced the use of the live infectious agent erysipelas (*S. pyogenes*) for the treatment of cancer. Since then, many bacterial strains have been investigated and tested in human patients (Table 5). Among the bacterial species selected for human studies, Listeria vaccine strains have demonstrated remarkably positive outcomes, and some strains are presently being tested in phase II and III clinical trials [209].

**Table 5:** Summary of studies conducted on clinical trials of oncolytic bacteria in cancer treatment

Bacterial strain	Clinical phase	Identifier no.	Type of cancer
*L. monocytogenes* (LADD)	Phase II	NCT01613313	Prostatic neoplasms
	Phase I	NCT02592967	Lung carcinoma
*L. monocytogenes*	Phase I	—	Metastatic pancreatic tumors
	Phase I	NCT00585845	Adenocarcinoma of the ovaries, pancreas, malignant epithelial mesothelioma, and
	Phase III	NCT02853604	Cervical cancer
*S. typhimurium* VNP20009	Phase I	NCT00004216	Advanced metastatic solid tumors
	Phase I	NCT00006254	Solid tumors
	Phase I	NCT00004988	Neoplasm metastatic tumor
Saltikva (*S. typhimurium* expresses human IL-2)	Phase II	NCT04589234	Metastatic pancreatic cancer
	Phase I	NCT01099631	Liver cancer
*S. typhimurium* VNP20009 expressing TAPET-CD (cytosine deaminase)	Phase I	—	Head and neck, and esophageal adenocarcinoma
*S. typhimurium* Ty21a VXM01	Phase I	—	Pancreatic cancer
*S. typhimurium* (χ4550)	Phase I	—	Hepatocellular carcinoma
*S. typhimurium* VNP20009	Phase I	—	Melanoma
*S. typhimurium* VNP20009	Phase I	—	Metastatic melanoma, metastatic RCC
*C. novyi*-NT	Phase Ib	NCT03435952	Refractory advanced solid tumors
	Phase I	NCT01924689	Solid tumor malignancies
	Phase I	NCT00358397	CRC
	Phase I	NCT01118819	Solid tumor malignancies
Mixed bacterial vaccine	Phase I	NCT00623831	Malignant tumors
*C. butyricum M55*	Phase I	—	Vascular glioblastoma

Despite the efforts made toward the clinical trials, only one (BCG) gained approval from the Food and Drug Administration (FDA) [30,163,210]. BCG is an attenuated strain of *Mycobacterium* with substantial merit as a treatment for bladder carcinoma (non-muscle invasive). BCG was approved in 1990 by the FDA and has been the benchmark therapeutic agent [26]. BCG therapy is employed by the administration of the BCG suspension via catheter into the bladder of the patients, with the mode of action remaining largely unclear. Nevertheless, it is known that direct contact with the malignant tissue is necessitated to promote an inflammatory response and a cytotoxic effect [211]. Regardless of the initial success of BCG, substantially few bacteriotherapy have reached the stage of clinical trials, especially in comparison to oncolytic viruses and nanoparticles. Currently, three bacterial species are at the forefront of clinical application: *S*. *typhimurium*, *C. novyi*-NT, and *L. monocytogenes*. These species have common characteristics with other bacteria and oncolytic viruses, including pre-clinical genetic engineering, displayed oncolytic effects in animal models, and tumor targeting potential naturally or artificially induced. Nevertheless, each of these species demonstrates a significantly distinct oncolytic mode of action [163,212–214].

Currently, intratumoral preparation of *C. novyi*-NT is entering a phase II clinical trial while simultaneously being explored in a phase I trial as a combinatorial therapy with pembrolizumab (anti-PD1 antibody) [215]. Pre-clinical data indicate that *C. novyi*-NT combination bacteriolytic therapy (COBALT) has powerful anticancer activity because of the contrasting cytotoxicity processes and remarkably precise innate targeting [216]. *C. novyi*-NT has completed a phase Ib clinical trial (NCT01924689) and is well-tolerated in solid tumor patients, which is significant progress for oncolytic bacteriotherapy [217].

*S*. *typhimurium* is also moving through the good pipeline of the clinical trial. At the forefront of *S*. *typhimurium* (strain Saltikva) investigation, the organism has been GM to induce to express IL-2, while the genes responsible for virulence factors have been knocked out [29]. A combination of this organism with an immunostimulatory cytokine is a continual direction in the field [218].

*L. monocytogenes* (ADXS11-001) is positioned to reach FDA approval. It is presently being explored in a phase III clinical trial for the cervical cancer (NCT02853604) treatment. *L. monocytogenes* was manipulated to be engulfed by APC cells and produced an antigen–adjuvant fusion protein in order to modify the TEM [219], facilitating T-cell infiltration and minimizing the intrinsic immune suppression properties of the TME [217,219]. Table 5 summarizes some of the major clinical trials of oncolytic bacteria reported, along with their findings.

## 9 Conclusions and future perspectives

Oncolytic bacteria are still a relatively unexplored cancer treatment approach but could be advantageous over conventional therapies such as chemotherapy and radiation. Various clinical trials have demonstrated that oncolytic bacteria have the unique ability to selectively infiltrate tumors, destroy tumor cells, and stimulate immune responses against cancer. Although their replication and lytic activity primarily target benign tumors, they can also impact metastases, making oncolytic bacteria a versatile and powerful tool in cancer treatment.

The future of oncolytic therapy holds immense promise. Advances in the introduction of new genomic tools will likely open the therapeutic avenue for different types of cancers that are not responsive to chemotherapeutic agents with acceptable levels of safety profiles. Researchers are working to minimize the different types of barriers, such as the complexity of the TME and its resistance to the oncolytic agent. Furthermore, the synergy of oncolytic bacteria with other conventional therapeutic approaches like immune checkpoint inhibitors or classical chemotherapy could enhance the efficiency of the treatment and expand the use of the approach across different types of cancers.

Another exciting opportunity in the rim of oncolytic therapy is the discovery of new microbial-based immunotherapy that will be customized depending on a patient’s genetic and immunological status. This could enhance the effects of the treatment while reducing side effects, at the same time, by identifying specific molecular weaknesses inherent in cancer patients. Moreover, the incorporation of imaging, biomarkers and other advanced techniques may offer an opportunity to monitor bacterial therapy outcomes in real-time and to modify the treatment accordingly. Further studies on the relationships occurring between oncolytic bacteria and the immune system of the host will subsequently reveal further findings showing the potential for more advanced and selective treatments for cancer.

In conclusion, despite the immense potential of oncolytic therapy, there are some problems and drawbacks, such as the lack of the best delivery methods for these bacteria into tumor tissues and the problems with possible off-target effects. Nevertheless, the continuous research conducted in this area and the constant advancement of biotechnological tools build an optimistic perspective for oncolytic therapy in the near future. Ongoing interprofessional cooperation and sustained commitment to translational research may turn bacterial oncolysis into a paradigm-shifting advancement in cancer treatment that will forge a better quality of life for patients fighting an intricate disease.
